# Voluntary Neonatal Medication Incident Reporting—A Single Centre Retrospective Analysis

**DOI:** 10.3390/healthcare12212132

**Published:** 2024-10-25

**Authors:** Sunaina Nundeekasen, Joanne McIntosh, Laurence McCleary, Cathryn O’Neill, Tejasvi Chaudhari, Mohamed E. Abdel-Latif

**Affiliations:** 1 Department of Neonatology, Centenary Hospital for Women and Children, The Canberra Hospital, Garran, ACT 2605, Australia; sunaina.nundeekasen@act.gov.au (S.N.); tejasvi.chaudhari@act.gov.au (T.C.); 2 Neonatal Intensive Care Unit, John Hunter Children’s Hospital, Newcastle, NSW 2305, Australia; joanne.mcintosh@health.nsw.gov.au; 3 School of Medicine and Public Health, College of Health, Medicine and Wellbeing, University of Newcastle, Newcastle, NSW 2308, Australia; 4 School of Women’s and Children’s Health, University of New South Wales, Kensington, NSW 2052, Australia; laurence.mccleary@health.nsw.gov.au; 5 Department of Paediatrics, Gosford Hospital, Gosford, NSW 2250, Australia; 6 Nursing and Midwifery Directorate, Centenary Hospital for Women and Children, The Canberra Hospital, Garran, ACT 2605, Australia; 7 Discipline of Neonatology, School of Medicine and Psychology, College of Health and Medicine, Australian National University, Acton, ACT 2600, Australia; 8 The Department of Public Health, La Trobe University, Bundoora, VIC 3083, Australia

**Keywords:** neonate, incident, reporting, medication

## Abstract

**Background**: Medication errors in neonatal intensive care units (NICUs) are prevalent, with dosage and prescription errors being the most common. **Aims**: To identify the common medication errors reported over twelve years using a voluntary, nonanonymous incident reporting system (RiskMan clinical incident reporting information system) at an Australian tertiary NICU. **Methods**: This was a single-centre cohort study conducted at a tertiary NICU. All medication-related incidents (errors) reported prospectively through the RiskMan online voluntary reporting database from January 2010 to December 2021 were included. The medication incidents were grouped into administration, prescription, pharmacy-related, and others, which included the remaining uncommon incidents. **Results**: Over the study period, 583 medication errors were reported, including administration-related (41.3%), prescription-related (24.5%), pharmacy-related (10.1%), and other errors (24%). Most incidents were reported by nursing and midwifery staff (77%) and pharmacists (17.5%). Most outcomes were minor or insignificant (98%), with only a few resulting in major or significant harm. There was one extreme incident that may have contributed to the death of a neonate and nine moderate incidents. **Conclusions**: Our results demonstrate that medication errors are common and highlight the need to support improvement initiatives and implement existing evidence-based interventions in routine practice to minimise medication errors in the NICU.

## 1. Introduction

Medication-related errors are a common cause of hospital adverse events associated with severe harm or even death [1,2]. This risk is amplified more in the neonatal population given the small dosing targets, weight and gestation-based dosing, immature renal and hepatic function, the frequent use of off-label medications, and the patient’s inability to detect errors themselves [3,4].

Medication errors are among the most common incidents reported in neonatal intensive care units (NICUs) [5,6].

A systematic review conducted by Alghamdi et al. on the prevalence and nature of medication errors and preventable adverse drug events in paediatric and neonatal intensive care settings revealed that in neonatal intensive care units, medication error rates ranged from 4 to 35.1 per 1000 patient days and 5.5 to 77.9 per 100 medication orders [7]. A study examining two hospitals in the US reported that neonates in the NICU experienced significantly greater medication errors and potential ADE rates (91 and 46 per 100 admissions, respectively) than neonates in other wards did (50 and 9 per 100 admissions, respectively) (*p* < 0.001 for both comparisons) [8]. The importance of rigorous safety measures around medication prescribing and administration is paramount, given the high frequency of medication errors.

Voluntary nonpunitive reporting systems have been shown to help collect information about adverse medical incidents and subsequent system changes and preventative strategies to improve and optimise patient safety [5].

This study aimed to determine the common neonatal medication incidents voluntarily reported in our unit over twelve years.

## 2. Materials and Methods

This study was a single-centre cohort study conducted at a tertiary NICU. This study included analysis of all medication-related incidents (errors) reported prospectively through an online voluntary reporting database (RiskMan clinical incident reporting information system) from 1 January 2010 to 31 December 2021 [9]. The incidents were related to neonatal care in the neonatal unit, postnatal ward, delivery suite, birth centre, and operating theatre. RiskMan is a voluntary and nonanonymous incident reporting database accessible to all staff at our hospital. The Centenary Hospital for Women and Children is based in Canberra, Australia. The maternity unit conducts approximately 3800 births per annum. The NICU admits up to 800 patients per year.

During the study period, medications were prescribed via a conventional paper system. The medications are usually prescribed by the registrar or fellow under the supervision of a neonatologist. A clinical pharmacist usually attends morning ward rounds and reviews all prescriptions. The pharmacist often cannot review prescriptions during the rest of the day. The RiskMan incidents were reviewed, and all medication-related incidents were included in this study based on data and free text information from each report. The events were categorised into broad medication incident types by a neonatologist (SN) with a discussion with a senior neonatologist (MEA) if required. Duplicate incidents were excluded.

The incidents were categorised into administration-related, prescription-related, pharmacy-related, and other types. The categories were further broken down into incorrect dosage, incorrect time/omitted dose, incorrect rate, incorrect medication/fluid, incorrect labelling, administration without a prescription, orders not signed for, medication not being sent, expired medication, extravasation injuries, fluid leaks, medication trough levels not being performed or other issues, including but not limited to pump malfunction and charts not received by the pharmacy.

Incidents related to neonatal intravenous fluids were also included in this analysis, given their common carriage of medications and their potential for serious harm with errors.

The incident reporter assigned the outcome rating for each event at the time of report entry into the RiskMan database. These ratings were reviewed and, if needed, amended by trained incident classifiers from the Clinical Safety and Quality Unit using standard definitions. The definitions of the outcome ratings are described in Table 1. These definitions were available to the reporter at the time of reporting. All incidents and control measures are discussed at monthly departmental quality and safety meetings.

## 3. Results

There were 3070 reported incidents analysed during the study period. Among these, 21 duplicates were excluded, resulting in 3049 events. Medication-related events made up 583 (19.1%) incidents. This equates to 0.13 medication incidents per day or one error per 7.43 days.

**Figure 1:**
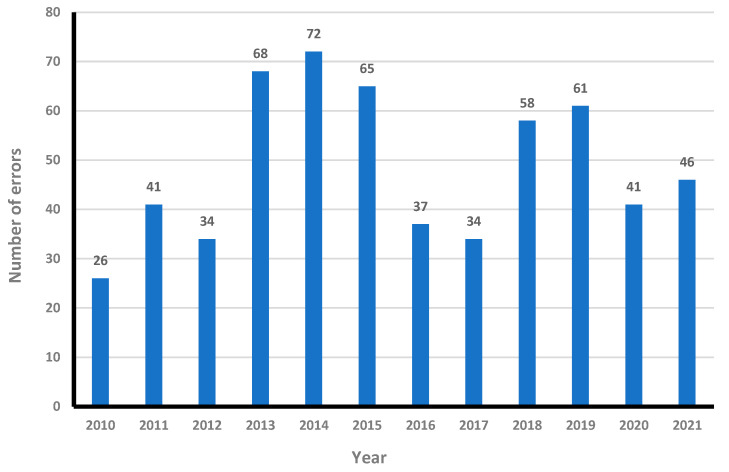
Number of medication errors by year.

Figure 1 shows the number of medication-related incidents by year. The most common medication incident was administration errors, followed by prescription and pharmacy-related errors (Table 2).

**Figure 2:**
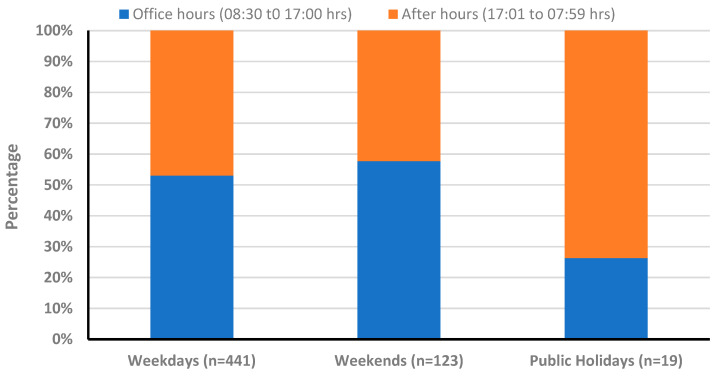
Number of medication-related incidents by time of day.

Figure 2 shows the number of medication-related incidents by time of day (the time was separated into 08:30 to 17:00 h, which correspond to office hours, and 17:01 to 08:29 h, which correspond to after-hours). Weekends and public holidays were divided in a similar way as there was a resident senior cover from 08:30 to 17:00 h). 

Table 3 details the medication incidents by category. Among administration-related errors (n = 241), incorrect time/omitted dose (n = 78; 32.3%) and incorrect rate (n = 59; 24.5%) were the most common groups.

Prescribing-related incidents made up 24.5% (n = 143) of the incidents, which included incorrect dosages (n = 57; 39.9%) and prescriptions not being signed for (n = 47; 32.9%), among others. Pharmacy-related incidents accounted for 10.1% (n = 59) of the incidents, 52.5% (n = 31) of which were incorrectly labelled medications (Table 3).

A total of 65 of the 143 prescription errors were related to vitamin K and hepatitis B prescriptions not being signed (n = 50), incorrect doses (n = 10), being incorrectly written (n = 3), and not being given a prescription (n = 2).

Most event outcomes were minor or insignificant (98.2% combined), with only 1.7% moderate or major (Table 4). One death was thought to be related to a medication incident; this was the only incident assigned an extreme rating (Table 5).

Most incidents were reported by nursing staff (77%), with 17.5% reported by a pharmacist. Only 2.5% of incidents were identified as being entered by a medical officer (Table 6).

## 4. Discussion

Medication errors in the neonatal setting are common, with dosage errors being the most common, as reported in multiple prior studies [8,10,11]. Our rate of 19.1% is similar to previously reported rates ranging from 4 to 28% [8,10].

There was minimal clinical impact of the medication errors in this study, likely because many errors were identified before harm occurred. Less than 2% of the errors were classified as major incidents. Similarly, Fernandez et al. [11] reported that potentially fatal or high-risk prescribing errors accounted for only 2% of the errors identified in their paediatric-based study. There was one serious incident in our study that may have contributed to the death of a neonate. The death resulted from a pericardial effusion secondary to total parenteral nutrition (TPN) given via a newly inserted percutaneous long line. This is not strictly a medication error and may be considered a device-related or iatrogenic error. Administration-related incidents, such as tenfold medication errors, were encountered (Table 5). These errors, though infrequent, can have severe consequences because of the vulnerability of neonatal patients. The complexity of neonatal pharmacotherapy, involving small volumes and precise dosing, increases the risk of such errors.

The rate of prescription errors was high in our study, with the majority being related to vitamin K and hepatitis B vaccination administration postbirth. Many of these incidents were related to errors such as signature omission. Previous studies have shown a similarly high rate of prescribing errors, ranging from 3 to 37% of medication error types [12].

Kaushal et al. [8] reported that NICU patients had a significantly higher rate of preventable adverse drug events than other wards did, highlighting the importance of utilising preventive strategies in this population to reduce the error rate. Many studies have examined this topic. Interventions previously studied include a clinical pharmacist [13,14,15,16], computerised physician order entry (CPOE) [17,18,19,20,21], bar code medication administration (BCMA), smart pumps, adequate training for doctors and nurses, and regular medication review [8,13,14,22,23].

Acheampong et al. [13] performed a systematic review of medication safety strategies, including those previously mentioned, and concluded that there is a lack of controlled studies for many interventions; thus, more studies are needed.

Nguyen et al. also conducted a systematic review to determine the effectiveness of interventions in reducing neonatal medication errors. They reported that no single intervention was superior and that multiple interventions to improve medication safety could reduce errors [24]. A recent QI project at a UK neonatal unit also revealed a significant reduction in medication errors following the implementation of multiple interventions by a project team comprising a neonatologist, a pharmacist, and nurses. These included regular staff teaching, drug prescribing tests, pharmacy quizzes, pharmacy-based simulations, sharing twice-monthly RED posters cross-site, sharing learning through weekly grand rounds and doctor and nursing handovers, and nursing tests and red aprons for nurses during drug administration [25]. Other strategies reported in the literature include electronic prescribing systems to minimise transcription errors and enhance staff education through regular training programs focused on dose calculations and error prevention [24,26].

Additionally, fostering a culture of safety where interdisciplinary teams collaborate and communicate effectively can significantly reduce errors. Risk management programs that involve routine review and feedback on medication practices have also been shown to lower error rates [14,27].

Studies have shown that despite various preventative interventions, no single strategy has proven superior in eliminating these errors [14,24]. Therefore, a multifaceted approach that includes technology, education, and organisational changes is essential to mitigate these risks effectively [26].

A survey of the clinical practices of 20 Australian and New Zealand level III neonatal units (NNUs) in the utilisation of neonatal medication error prevention strategies revealed that the evidence-based strategies most utilised were the use of smart pumps (n = 18; 90%) and ward-based clinical pharmacists (n = 17; 85%). The total number of evidence-based medication error prevention strategies utilised in each NNU ranged from 2 to 10 (median = 7), and 10 of 16 strategies were utilised by fewer than 50% of the NNUs [28].

Voluntary reporting systems, such as the one from which we have gathered data for this study, are known to underestimate the total number of events that occur [29]. These reporting systems have been shown to mainly report events where no harm occurred, with interventions occurring (such as a pharmacist alerting someone to an error) before the potentially harmful event. Snijders et al. [5] looked at incident reporting systems in neonatal intensive care units and concluded that voluntary nonpunitive reporting systems elicit more incidents than mandatory reporting systems do. Conversely, there have been suggestions that people are deterred from reporting incidents in a nonanonymous system, given the potential for disciplinary action secondary to adverse events.

Ligi et al. [30] looked at their anonymous voluntary nonpunitive incident reporting system in the NICU, analysing all iatrogenic events before and after multiple system changes were implemented to reduce these events and develop preventative initiatives. They reported a reduced number of iatrogenic events post-system changes, including a reduced number of drug errors. One of the main contributing reasons was thought to be a change in their policy and practice, such that handwritten prescriptions are only allowed for emergencies, thereby reducing the rate of dosing errors and any misreading of handwritten scripts. They also reported that their team was motivated to report incidents to improve patient care.

Ahluwalia and Marriott [31] described critical incident reporting systems and noted that it is vital to have a culture where this system is seen as a positive tool to improve the quality of health care, which is supported at the highest levels of the organisation, whereby feedback for serious events can be given, and recommendations for improvement can be made.

In our study, most incidents were reported by nursing staff, similar to the findings of Snijders et al. [5], who reported that 88% of events were reported by nursing staff. The reasons for the low frequency of medical officer reports are unclear. This may reflect the team’s local culture, i.e., the bedside nurse or pharmacist reports on behalf of the broader team or that medical staff lack time and familiarity with using the reporting system, or other reasons.

The limitations of our study include that it was a single-centre study reviewing incidents from a voluntary reporting system, which is likely to have underestimated the actual incident numbers within the NICU. Although the data collection and severity classification were prospective, the analysis and categorisation of incidents were conducted retrospectively. The strengths of this study include a large sample size over twelve years, detailed information that assisted in the analyses of most incidents and incident reporting from all healthcare providers, and prospective reporting and rating.

## 5. Conclusions

Our results demonstrate that medication errors are common and highlight the need to support improvement initiatives and implement existing evidence-based interventions in routine practice to minimise medication errors in the NICU. 

Future studies could include examining the merits of an anonymous reporting database and comparing reported events with actual event numbers on medical record reviews. Further local studies on the medication error rate after introducing preventative strategies such as electronic prescribing would also be helpful.

## Figures and Tables

**Table 1:** Incident outcome ratings used at Canberra Hospital.

Outcome Rating	Clinical Definition
Insignificant	No injury No review required No increased level of care
Minor	Minor injury requiring: ◦ Review and evaluation ◦ Additional observations ◦ First aid treatment
Moderate	Temporary loss of function (sensory, motor, physiological, or intellectual) unrelated to the natural course of the underlying illness and differing from the expected outcome of patient management. The incident resulted in the transfer to a higher level of care or additional procedure.
Major	Major and permanent loss of function (sensory, motor, physiological, or intellectual) unrelated to the natural course of the underlying illness and differing from the expected outcome of patient management.
Extreme, Catastrophic or Significant	All national core sentinel events *

Adapted from ACT Health Incident Management Policy and Procedures, 2012. * Core Sentinel events can be found in the Australian Commission on Safety and Quality in Healthcare 2008; National Safety and Quality Health Service Standards, Commonwealth of Australia.

**Table 2:** Medication incidents by category.

Incident Category	Number (%)
Administration error	241 (41.3)
Prescribing error	143 (24.5)
Dispensing error	59 (10.1)
Others *	140 (24)
Total	583 (100)

The data are presented as numbers (%). * Others include extravasations, fluid disconnection, incorrect patient labels, equipment failures, missed drug levels, and missed documentation for indications for antibiotic treatment.

**Table 3:** Details of medication incidents by category.

Error	Administration Related (n = 241)	Prescription Related (n = 143)	Dispensing Related (n = 59)	Others (n = 140)	Total (n = 583)
Incorrect dose	29 (12.1)	57 (39.9)	15 (25.4)	0	101 (17.3)
Incorrect time/omitted dose	78 (32.4)	26 (18.2)	1 (1.7)	0	105 (18)
Incorrect rate	59 (24.5)	2 (1.4)	0	0	61 (10.5)
Incorrect medication/fluid	50 (20.7)	11 (7.7)	6 (10.2)	0	67 (11.5)
Incorrect label	3 (1.2)	0	31 (52.5)	27 (19.3)	61 (10.5)
Given without prescription	15 (6.2)	0	0	0	15 (2.6)
Not signed	7 (2.9)	47 (32.9)	0	0	54 (9.3)
Medication not sent	0	0	5 (8.5)	0	5 (0.9)
Expired medication	0	0	1 (1.7)	6 (4.3)	7 (1.2)
Extravasation	0	0	0	50 (35.7)	50 8.6)
Leak	0	0	0	10 (7.1)	10 (1.7)
Drug level not done	0	0	0	14 (10)	14 (2.4)
Others *	0	0	0	33 (23.6)	33 (5.7)

The data are presented as numbers (%). * Others include electronic issues, pump failures, milk in the wrong pot, etc.

**Table 4:** Number of events and examples per outcome rating.

Outcome Rating	Example from the Database	Number (%)
Insignificant	Missed gentamicin level	360 (61.7)
Minor	Vitamin dose given in error	213 (36.5)
Moderate	Medication overdose	9 (1.5)
Extreme, Catastrophic or Significant	Pericardial effusion secondary to total parenteral nutrition given via the wrongly positioned central line.	1 (0.2)
Grand Total	- - -	**583 (100%)**

The data are presented as numbers (%).

**Table 5:** List and brief descriptions of medication incidents with moderate-to-extreme outcome ratings (n = 10). TPN denotes total parenteral nutrition.

Gestation and Age	Brief Description	Outcome
A male infant born at 29 weeks gestation. The error occurred at 39 weeks CGA.	The baby was given two times the dose of thiopentone and, therefore, remained ventilated for longer than necessary.	Moderate ^1^
Male born at 25 weeks gestation. Error on day 2 of life.	The baby was administered ten times the dose of morphine as an intravenous stat bolus. The baby had severe unrelated comorbidities and passed away on the same day from an unrelated cause.	Moderate ^1^
Male born at 28 weeks gestation. Error at 40 weeks CGA.	The baby was given four times the dose of dexamethasone as drawn up directly from the vial rather than from the diluted syringe. This led to a rise in blood pressure, which requires antihypertensives.	Moderate ^1^
Male infant born at 40 weeks gestation. Error on day 1 of life.	The baby was given a double dose of gentamicin. A referral was made for a hearing assessment due to gentamicin toxicity.	Moderate ^1^
Female 33 weeks gestation. Error day 1 of life.	Gentamicin was given 12 hourly instead of 24 hourly. A referral was made for a hearing assessment.	Moderate ^2^
Female born at 25 weeks gestation. Error at 28 weeks CGA.	A tailored bag of total parenteral nutrition (TPN) was made up in the local pharmacy with 20% sodium chloride instead of 0.9% sodium chloride. This led to hypernatraemia with a serum sodium rise from 133 to 160.	Moderate ^3^
Male born at 39 weeks gestation. An error occurred on day 2 of life.	The baby received ten times the dose of morphine, leading to hypotension requiring inotropes.	Moderate ^1^
Male born at 34 weeks gestation. The error occurred at one week of life.	The lipid infusion line was found to be disconnected from the peripherally inserted intravenous cannula in the isolette. This was reconnected instead of a new sterile bag of TPN and lipids. The baby had a positive blood culture a few days later.	Moderate ^4^
Male born at 30 weeks gestation. An error occurred on day 2 of life.	Gentamicin was given 12 hourly instead of 36 hourly. A referral for a hearing assessment was made due to potential gentamicin toxicity.	Moderate ^2^
Male infant born at 28 weeks gestation. Error at 11 days of life.	A peripheral, central line was inserted, and the tip was seen to be in the right heart. The line was readjusted, and an X-ray showed a tip in the superior vena cava. The line was connected to TPN. The baby suddenly deteriorated and had cardiac arrest requiring extensive resuscitation. Postmortem showed a 30 mL pericardial effusion consistent with TPN.	Extreme/Catastrophic/Significant ^4^

The medication incident categories included ^1^ administration-related, ^2^ prescription-related, ^3^ dispensing-related, and ^4^ others.

**Table 6:** Reporter identification on the voluntary incident reporting system (RiskMan) database for medication incidents.

Reporter	Number (%)
Registered nurse/midwife	449 (77.0)
Pharmacist	102 (17.5)
Other health professional *	11 (1.8)
Consultant	3 (0.5)
Junior medical officer	12 (2.0)
Not identified	6 (1.1)
Grand total	**583 (100)**

The data are presented as numbers (%). * Other health professional: professional health worker not identifiable with other groups provided (e.g., physiotherapist, dietician).

## Data Availability

The data may be obtained from a third party and are not publicly available. The data for this study were extracted from the Incident Management Database (RiskMan), an ongoing prospective audit of all clinical incidents. The authors will not be able to share any individual participant data. However, the data may be available upon reasonable request from The Incident Management Team; Quality, Safety, Innovation and Improvement; Canberra Health Services; Australia. All data relevant to the study are included in this article.
